# A Dynamic and Complex Network Regulates the Heterosis of Yield-Correlated Traits in Rapeseed (*Brassica napus* L.)

**DOI:** 10.1371/journal.pone.0021645

**Published:** 2011-07-01

**Authors:** Jiaqin Shi, Ruiyuan Li, Jun Zou, Yan Long, Jinling Meng

**Affiliations:** National Key Laboratory of Crop Genetic Improvement, Huazhong Agricultural University, Wuhan, Hubei, China; Lund University, Sweden

## Abstract

Although much research has been conducted, the genetic architecture of heterosis remains ambiguous. To unravel the genetic architecture of heterosis, a reconstructed F_2_ population was produced by random intercross among 202 lines of a double haploid population in rapeseed *(Brassica napus* L.). Both populations were planted in three environments and 15 yield-correlated traits were measured, and only seed yield and eight yield-correlated traits showed significant mid-parent heterosis, with the mean ranging from 8.7% (branch number) to 31.4% (seed yield). Hundreds of QTL and epistatic interactions were identified for the 15 yield-correlated traits, involving numerous variable loci with moderate effect, genome-wide distribution and obvious hotspots. All kinds of mode-of-inheritance of QTL (additive, A; partial-dominant, PD; full-dominant, D; over-dominant, OD) and epistatic interactions (additive × additive, AA; additive × dominant/dominant × additive, AD/DA; dominant × dominant, DD) were observed and epistasis, especially AA epistasis, seemed to be the major genetic basis of heterosis in rapeseed. Consistent with the low correlation between marker heterozygosity and mid-parent heterosis/hybrid performance, a considerable proportion of dominant and DD epistatic effects were negative, indicating heterozygosity was not always advantageous for heterosis/hybrid performance. The implications of our results on evolution and crop breeding are discussed.

## Introduction

Heterosis is defined as the superior performance of crossbred characteristics as compared with corresponding inbred ones [1]. The utilization of heterosis has become a major strategy to increase the productivity of plants and animals [2]. Despite the successful utilization of heterosis in many crops, there still exists a contradiction between the agricultural practice of heterosis utilization and our understanding of the genetic basis of heterosis and this hampers the effective exploitation of this biological phenomenon [3].

The classical quantitative genetic explanation of heterosis centered on three hypotheses: dominance, over-dominance and epistasis [4], [5]. Evidence of these genetic models remained unavailable until very recent advances in molecular marker technology, high-density linkage maps and genome sequencing. Although much research into the genetic basis of heterosis in crops and plants has been conducted, little consensus has emerged. Research has indicated that heterosis may be attributable to dominance, over-dominance, epistasis or a combination of all of these, depending on the study materials, traits and analytical approach. Typically, little is known about the genetic control of heterosis in the complex polyploid crop rapeseed (*Brasscia napus* L.). Based on the phenotype of the E×R53-DH population and the corresponding BC population, as well as the mid-parent heterosis of the BC population, Radoev et al. (2008) mapped 33 QTL (9 of which showed a significant dominant effect) and a large number of epistatic interactions for seed yield and the three yield-component traits. They concluded that epistasis together with all levels of dominance from partial to over-dominance is responsible for the expression of heterosis in rapeseed [6]. Based on this E×R53-DH population and another E×V8-DH population with the same parent, and using the same experimental design, Basunanda et al. (2010) detected a number of QTL hotspots responsible for seedling biomass and yield-related traits. Given the key role of epistatic interactions in the expression of heterosis in oilseed rape, they supposed that these QTL hotspots might harbour genes involved in regulation of heterosis for different traits throughout the plant life cycle, including a significant overall influence on heterosis for seed yield [7]. However, in both studies, all kinds of genetic effects (A, D and AA, AD/DA, DD) were unable to be estimated in the same population, thus it was difficult to accurately estimate their mode-of-inheritance and relative importance in the expression of heterosis.

There were several common patterns described in most of these studies. Firstly, the QTL for yield and yield-correlated traits tended to be clustered in the genome in many crop and model plants, such as rice [8], maize [9], wheat [10], rapeseed [7] and *Arabidopsis* [11], which suggested the QTL of yield-correlated traits might have pleiotropic effects. However, this kind of pleiotropy has not been well analyzed genetically. Secondly, only a few limited traits were investigated and only a few QTL and epistatic interactions were identified for each trait, so a relatively comprehensive picture of the genetic architecture of heterosis remained unavailable. Thirdly, trials were carried out in only one or two environments and the environmental response of QTL and epistatic interactions for heterosis was not analyzed and thus remains unclear.

The main objective of this study was to unravel the genetic architecture of heterosis with QTL mapping in rapeseed, including: (1) determine the level of heterosis for a range of yield-correlated traits; (2) investigate the relationship between molecular marker heterozygosity and heterosis/hybrid performance; (3) identify QTL and epistatic interactions underlying heterosis and estimate their genetic effect, mode-of-inheritance and environmental responses; (4) analyze the relative contribution of all kinds of genetic effects in the expression of heterosis in rapeseed (*Brassica napus* L.).

## Results

### Correlation of trait performance and mid-parent heterosis among the 15 investigated traits

In the same environment, most pair-wise genetic correlations of performance and mid-parent heterosis were similar (Table S1A–C). This was understandable since mid-parent heterosis was calculated from trait performance. In different environments, pair-wise genetic correlations differed considerably (mostly in degree, a few in direction), which suggested that genetic correlations depended strongly on the environments.

Genetic correlations of performance and mid-parent heterosis among the investigated traits were also calculated across the three environments (Table 1). In general, significant correlations were observed for 81.9% and 67.6% of the pair-wise combinations of the trait performance and mid-parent heterosis, respectively. Seed yield correlated significantly with the other 14 investigated traits for both trait performance and mid-parent heterosis; negatively for flowering time, maturity time and protein content, and positively for the other 11 ones. Interestingly, the mean *r* ^2^ of trait performance was somewhat higher than that of mid-parent heterosis for most traits, ranging from 0.04 and 0.03 (for seed development times) to 0.24 and 0.20 (for seed yield), respectively.

**Table 1:** Genetic correlations of trait performance (above diagonal) and mid-parent heterosis (below diagonal) among the 15 investigated traits across three environments.

Trait§	BN	BY	DT	FT	HI	MT	OIL	PH	PN	PRO	PY	SN	SP	SW	SY	Mean r^2^
BN		0.32‡	-0.10*	-0.17*	0.23‡	-0.26‡	0.09	0.37‡	0.35‡	-0.17*	0.06	0.11*	0.40‡	-0.07	0.39‡	0.06
BY	0.36‡		0.01	0.05	-0.09	0.02	0.10*	0.64‡	0.58‡	-0.04	0.19†	0.12*	0.60‡	0.08	0.69‡	0.13
DT	-0.05	0.02		-0.50‡	0.00	0.41‡	-0.09	-0.11*	-0.04	0.23‡	0.09	-0.09	-0.11*	0.27‡	0.19†	0.04
FT	-0.11*	-0.09	-0.46‡		-0.52‡	0.55‡	-0.08	0.03	-0.05	0.21‡	-0.27‡	-0.16*	-0.15*	-0.18†	-0.26‡	0.08
HI	0.20†	-0.08	0.04	-0.25‡		-0.54‡	0.38‡	0.12*	0.37‡	-0.48‡	0.37‡	0.44‡	0.62‡	-0.12*	0.64‡	0.16
MT	-0.16*	-0.05	0.53‡	0.31‡	-0.31‡		-0.18†	-0.08	-0.13*	0.44‡	-0.18†	-0.25‡	-0.30‡	0.11*	-0.28‡	0.10
OIL	0.02	-0.01	0.01	-0.12*	0.31‡	-0.23‡		0.22‡	0.17*	-0.38‡	0.19†	0.35‡	0.39‡	-0.23‡	0.33‡	0.06
PH	0.45‡	0.59‡	-0.02	-0.08	0.07	-0.14*	0.07		0.39‡	-0.22‡	0.22‡	0.22‡	0.51‡	-0.01	0.56‡	0.11
PN	0.31‡	0.57‡	0.00	-0.11*	0.33‡	-0.15*	0.11*	0.32‡		-0.21‡	-0.32‡	-0.18†	0.73‡	-0.25‡	0.69‡	0.15
PRO	-0.09	0.00	0.13*	0.16*	-0.25‡	0.37‡	-0.45‡	-0.12*	-0.12*		-0.10*	-0.28‡	-0.38‡	0.26‡	-0.30‡	0.08
PY	0.07	0.17*	0.00	-0.06	0.22†	-0.02	0.04	0.14*	-0.40‡	0.02		0.78‡	0.22‡	0.36‡	0.42‡	0.10
SN	0.07	0.07	-0.09	-0.02	0.25‡	-0.10	0.04	0.11*	-0.36‡	-0.12*	0.86‡		0.49‡	-0.29‡	0.41‡	0.12
SP	0.39‡	0.64‡	-0.07	-0.16*	0.58‡	-0.25‡	0.16*	0.43‡	0.70‡	-0.24‡	0.16*	0.31‡		-0.41‡	0.90‡	0.23
SW	-0.02	0.15*	0.20†	-0.07	-0.08	0.19*	-0.05	0.05	-0.14*	0.28‡	0.29‡	-0.22†	-0.29‡		0.20†	0.05
SY	0.40‡	0.73‡	0.11*	-0.20†	0.58‡	-0.20†	0.16*	0.46‡	0.68‡	-0.14*	0.28‡	0.24‡	0.93‡	0.17*		0.24
Mean r^2^	0.06	0.13	0.03	0.04	0.09	0.06	0.04	0.08	0.14	0.05	0.09	0.09	0.19	0.04	0.20	

§The abbreviation of the traits, see MATERIALS AND METHODS.

*, ^†^ and ^‡^represent the significant level of P = 0.05, 0.01 and 0.001 respectively.

### Traits showing significant heterosis

The analysis of variance (in both populations) revealed that genotype, environment and the interaction between them had significant effect on the performance of all the 15 yield-correlated traits (Table S2A), so they were calculated separately for each environment. The broad-sense heritability of these traits ranged from 0.58 (for seed yield) to 0.90 (for flowering time), with a mean of 0.73. The two parents showed significant differences in 38 of the 43 trait-environment combinations (Table S2B). The two populations showed obvious transgressive variation for all of the trait-environment combinations. It should be noted that DH and the reconstructed-F_2_ population showed over-F_1_ variations for 13 (except seed yield and seed number per plant) and all of the traits respectively in all environments, which indicated that heterozygosity was not always favorable for trait performance. There was significant heterosis on F_1_ and F_2_ generations compared with the mean of the parents and the DH population, respectively, for the nine (branch number, biomass yield, harvest index, plant height, pod number, pod yield, seed number per pod, seed number per plant and seed yield) and eight (except branch number) traits. Interestingly, for these traits with significant heterosis, the performance of F_1_ was significantly higher than the mean of the F_2_ population and higher than the mean of the DH population in 19 and all of the 25 trait-environment combinations respectively, which showed an obvious trend of inbreeding depression.

According to the significance of heterosis, the 15 yield-correlated traits could be classified into two groups: the nine traits (seed yield, seed number per plant, biomass yield, pod number, harvest index, plant height, pod yield, seed number per pod and branch number) with heterosis and the other six traits (oil content, protein content, maturity time, flowering time, seed weight and seed development time) without heterosis. It should be noted that the correlation coefficients between seed yield and the nine traits with heterosis were all higher than that between the other six traits without heterosis.

The analysis of variance revealed that genotype, environment and genotype × environment interaction had significant effect on mid-parent heterosis of the nine traits with heterosis (Table S2C), so they were calculated separately for each environment (Table 2). For hybrid F_1_, seed yield and seed number per plant showed strong mid-parent heterosis, biomass yield and pod number per plant showed moderate mid-parent heterosis, while pod yield, seed number per pod, harvest index, branch number and plant height showed low mid-parent heterosis. For the reconstructed F_2_ population, the amount of heterosis varied widely for these traits, from highly negative to highly positive. The average mid-parent heterosis of the reconstructed F_2_ population showed similar trend with that of F_1_ for the nine traits. It should be noted that in each environment the mid-parent heterosis of some (the proportion is 10.2% for seed yield in S5 environment, data not shown) combinations of reconstructed F_2_ population was higher than that of F_1_, but the average mid-parent heterosis in the reconstructed F_2_ population was in all cases lower than that in F_1_. This indicated that heterosis was generally related to the heterozygosity at the population level but poorly correlated with heterozygosity at the individual level.

**Table 2:** Mid-parent heterosis of F_1_ and reconstructed F_2_ population in three environments for the nine yield-correlated traits with significant heterosis.

Traits§	Environments	Mid-parent heterosis					
		F_1_		reconstructed F_2_			
		value %		Mean %		range %	
SY	N6	1784	75.4	666	29.4	-854—2318	-32.2—126.2
	S5	866	69.7	403	30.5	-763—1143	-31.5—101.5
	S6	1026	99	309	28.5	-378—1104	-31.3—133.4
SP	N6	5057	67.3	2290	33.4	-2088—5803	-19.1—111.7
	S5	2714	70.4	1163	26.5	-2291—3964	-34.2—89.5
	S6	2577	84.8	859	26.6	-1296—3606	-27.9—99.1
BY	N6	2151	47.5	976	21.4	-1272—3354	-19.7—81.2
	S6	1329	40.5	688	19.6	-1254—2571	-37.3—87
PN	N6	179	53.7	61	20.2	-134—340	-30.8—82.5
	S5	79	29.6	42	16.9	-122—228	-34.2—79.5
	S6	106	33.1	39	18.2	-109—308	-27.8—80.6
HI	N6	5	14.6	2.3	9.1	-3.5—9.5	-9.1—39.5
	S6	6	24.8	3.7	14.1	-4.1—10.6	-7.3—52.2
PH	N6	20.6	17.7	13.6	11.2	-6.6—41.3	-5.7—36.1
	S5	18.4	13.6	8.5	6.1	-17.7—28.7	-13.4—20.1
	S6	12.3	9.4	8.6	6.4	-14.3—31.9	-9.3—26
PY	N6	1.1	15.2	0.56	8.8	-3.03—3.61	-18.5—49.4
	S5	1.6	31.5	0.48	10.1	-1.73—2.87	-16.9—43.7
	S6	1	21.9	0.42	9.4	-1.93—3.19	-27.4—57.1
SN	N6	5	21.8	2.2	11.8	-8.6—11.6	-35.8—72.0
	S5	3.7	23	1.5	10	-5.3—7	-29.9—47.1
	S6	2.7	20.6	1.2	8.6	-5.0—8.2	-33.9—62.5
BN	N6	1.34	16	0.9	10.5	-2.44—4.33	-27.3—64.8
	S5	1.52	21.6	0.53	7.7	-1.82—2.9	-27.4—45.4
	S6	0.62	11.3	0.31	5.6	-2.03—2.4	-35.2—43.6

§For the abbreviation of the traits (ordered according to their correlation coefficients with SY), see MATERIALS AND METHODS.

It should be noted that, for these yield-correlated traits, the heritabilities (ranging from 0.40 to 0.60) of mid-parent heterosis were all lower than that (ranging from 0.58 to 0.90) of trait performance (Table S2A; Table S2C).

### Correlation between heterozygosity and hybrid performance/mid-parent heterosis for the nine traits with significant heterosis

The correlation between heterozygosity and hybrid performance/mid-parent heterosis was significant for the nine traits with significant heterosis except branch number and seed number per pod (Table 3), with mean *r* ^2^ ranging from 0.001 (branch number) to 0.066 (seed yield) for the different traits, which accorded well with the heterosis level of these traits. Generally, the mean *r* ^2^ between heterozygosity and hybrid performance was similar to that between heterozygosity and mid-parent heterosis. Whereas, the mean *r* ^2^ (0.026/0.022) between special heterozygosity and hybrid performance/mid-parent heterosis was a little higher than that (0.013/0.014) of general heterozygosity and hybrid performance/mid-parent heterosis in most cases. Interestingly, the mean *r* ^2^ between heterozygosity and hybrid performance/mid-parent heterosis was stronger in the S5 environment than in the other two environments, which suggested these correlations were also depended on the environment. Although 47 of the 100 correlations between heterozygosity and hybrid performance/mid-parent heterosis were significant, the *r* ^2^ were relatively small (from 1.21% to 18.5%), which suggested that molecular marker heterozygosity could not predict hybrid performance and mid-parent heterosis.

**Table 3:** Correlations between general heterozygosity/special heterozygosity and hybrid performance/mid-parent heterosis in three environments for the nine yield-correlated traits with significant heterosis.

Traits§			SY	SP	BY	PN	HI	PH	PY	SN	BN	Mean r^2^
Hybrid performance	General heterozygosity	N6	0.18*	0.15*	0.11*	0.10*	0.13*	0.08	0.09	0.06	-0.03	0.012
		S5	0.30‡	0.18*	/	0.12*	/	0.09	0.11*	0.01	0.04	0.022
		S6	0.13*	0.09	0.10*	0.06	0.08	0.02	0.07	0.04	-0.01	0.006
		Mean *r* ^2^	0.046	0.021	0.011	0.009	0.011	0.005	0.009	0.002	0.001	0.013
	Special heterozygosity	N6	0.24‡	0.23‡	0.09	0.19*	0.17*	0.07	0.08	0.07	-0.01	0.022
		S5	0.43‡	0.29‡	/	0.17*	/	0.11*	0.17*	0.09	0.08	0.05
		S6	0.22†	0.09	0.09	0.14*	0.07	-0.07	0.04	0.09	-0.06	0.012
		Mean *r* ^2^	0.096	0.048	0.008	0.027	0.017	0.007	0.013	0.007	0.003	0.026
Mid-parent heterosis	General heterozygosity	N6	0.16*	0.13*	0.08	0.06	0.19*	0.08	0.12*	0.06	0.01	0.012
		S5	0.29‡	0.20†	/	0.11*	/	0.13*	0.13*	0.05	0.01	0.025
		S6	0.15*	0.11*	0.08	0.08	0.12*	0.05	0.06	0.02	-0.01	0.008
		Mean *r* ^2^	0.045	0.023	0.006	0.007	0.024	0.009	0.012	0.002	0.000	0.014
	Special heterozygosity	N6	0.21†	0.19*	0.02	0.12*	0.19*	0.07	0.13*	0.04	0.01	0.017
		S5	0.39‡	0.27‡	/	0.16*	/	0.16*	0.14*	0.09	0.01	0.044
		S6	0.19*	0.07	0.06	0.12*	0.11*	0.00	0.04	0.04	-0.02	0.008
		Mean *r* ^2^	0.078	0.038	0.002	0.019	0.025	0.010	0.013	0.004	0.000	0.022
Mean *r* ^2^			0.066	0.032	0.007	0.016	0.019	0.008	0.012	0.004	0.001	0.019

§ For the abbreviation of the traits (ordered according to their correlation coefficients with SY), see MATERIALS AND METHODS.

*, ^†^ and ^‡^ represent the significant level of P = 0.05, 01 and 0.001 respectively**.**

### Genome-wide detection and meta-analysis of QTL for 15 yield-correlated traits

**Figure 1:**
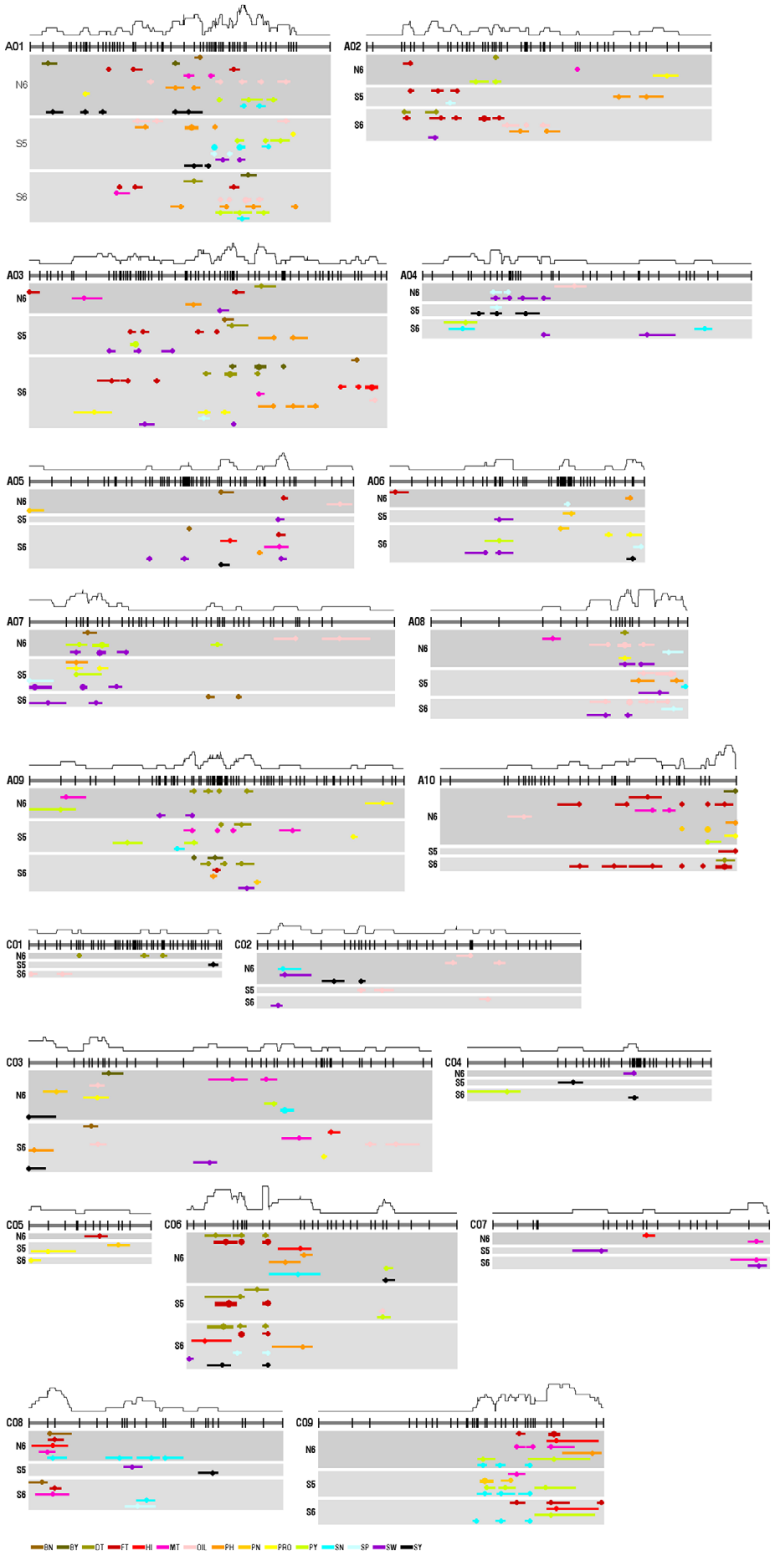
The genome-wide distribution of QTL identified in reconstructed F_2_ population and three environments for 15 yield-correlated traits. A total of 390 QTL were identified in reconstructed F_2_ population and three environments for 15 yield-correlated traits. The 19 linkage groups of TNDH linkage map are shown as a thick black line with vertical lines to indicate the position of the molecular markers, and the labels on the left represent their name (A genome: A01-A10; C genome: C01-C09). Under the linkage group lines, the QTL are drawn with horizontal bars where their lengths show the confidence interval, the circle indicates the peak position and the width of the QTL line imply the magnitude of their phenotypic variance (*R* ^2^<10%; 10%≤*R* ^2^<20%; 20%≤*R* ^2^<30%), and the labels on the left represent the codes of the three environments (N6, S5, S6) in which these QTL was identified. Above these linkage group lines, the black curves indicate the frequency of distribution of QTL. At the bottom of the figure, the horizontal lines of different colour indicate the different traits, and the letters on the right represent their abbreviations (see MATERIALS AND METHODS).

A total of 967 QTL (579 significant QTL and 388 suggestive QTL) were identified for the 15 yield-correlated traits in both populations in three environments (Table S3A). Exclusion of 209 non-overlapping suggestive ones, a total of 758 QTL was identified finally. Of which 390 identified QTL were from reconstructed F_2_ population (ranging from 11 to 56 for each trait) (Table 4; Figure 1), they were potentially responsible for heterosis and were the objectives of the following analysis. The 390 identified QTL explained 1.4-20.8% (mean = 5.6%) of the phenotypic variance while 92.8% showed only moderate effect, with *R* ^2^<10% and only one explained > 20% of phenotypic variance (Table S3B). Furthermore, for the 13 identified QTL with *R* ^2^≥10%, the absolute values of their dominant degree (∣D/A∣) were all < 1. This suggested that heterosis of these yield-correlated traits was typically controlled by numerous loci with little heterotic effect.

**Table 4:** Overview of identified and consensus QTL for 15 yield-correlated traits.

Trait	SY§	SP	BY	PN	HI	PH	PY	SN	BN	OIL	PRO	MT	FT	SW	DT	Total
Identified QTL																
Total number	23	15	10	10	11	31	32	27	11	43	18	26	56	46	31	390
significant level	17	14	9	7	9	22	24	22	10	33	12	19	47	32	27	304
suggestive level	6	1	1	3	2	9	8	5	1	10	6	7	9	14	4	86
Mean	7.7	5.0	5.0	3.3	5.5	10.3	10.7	9.0	3.7	14.3	6.0	8.7	18.7	15.3	10.3	9.1
*R* ^2^ min (%)	3.4	1.7	3.9	3.5	2.3	3.0	1.4	3.2	3.8	2.0	2.9	2.3	2.2	2.4	2.7	1.4
*R* ^2^ max (%)	7.9	9.8	11.0	12.2	19.9	11.0	11.8	12.4	7.2	12.8	8.1	9.1	20.8	18.4	10.9	20.8
*R* ^2^ mean (%)	5.5	4.4	6.4	6.8	6.1	5.3	4.6	6.1	4.9	6.2	5.6	5.3	6.1	5.6	5.7	5.6
Sum *R* ^2^ mean (%)	42.3	21.9	32.0	22.5	33.8	55.3	48.5	54.7	18.0	88.8	33.4	46.2	114.3	85.8	58.8	51.2
Additive-effect direction (+/-)	12-	6-	4-	3-	6-	14-	18-	13-	6-	17-	9-	8-	9-	30-	21-	176
	11+	9+	6+	7+	5+	17+	14+	14+	5+	26+	9+	18+	47+	16+	10+	214
Dominant-effect direction (+/-)	5-	3-	6-	5-	2-	15-	14-	9-	6-	15-	6-	12-	34-	19-	12-	163
	18+	12+	4+	5+	9+	16+	18+	18+	5+	28+	12+	14+	22+	27+	19+	227
Overlapped	4	4	0	2	2	10	18	15	0	21	2	6	41	22	20	167
Mean |D|/Mean |A|	0.73	0.68	0.50	0.48	0.46	0.43	0.60	0.44	0.40	0.45	0.53	0.49	0.42	0.55	0.53	0.51
Consensus QTL																
Total number	21	13	10	9	10	26	22	18	11	32	17	23	34	35	19	300
Mean	7.0	4.3	5.0	3.0	5.0	8.7	7.3	6.0	3.7	10.7	5.7	7.7	11.3	11.7	6.3	7.0
Repeatable	2	2	0	1	1	5	8	6	0	10	1	3	19	11	8	77

§ For the abbreviation of the traits (ordered according to their correlation coefficients with SY), see MATERIALS AND METHODS.

* Additative-effect direction (+/-).

‡ Dominant-effect direction (+/-).

† Mean |D|/Mean |A|.

To estimate the environmental response of QTL in natural environments, meta-analysis was used to integrate the identified QTL trait-by-trait in different environments (Table 4; Table S3C). A total of 300 consensus QTL was identified, of which only 77 (25.7%) were repeatedly found in more than two environments and regarded as repeatable QTL, the other 223 (74.3%) were specifically identified in one of the three environments and considered as non-repeatable ones (Table 5). This indicated that the expression of QTL of yield-correlated traits was strongly dependent on environmental conditions, which is also confirmed by the result that 55.3% (166/300) of consensus QTL showed significant QTL × environment interaction in ANOVA analysis (Table S3C). The proportion of the repeatable QTL was high for flowering time, development time of seeds, pod yield and seed number per pod, and results accorded with the high heritability of these traits. Only 77 consensus QTL were repeatable, whereas 68.8% changed their mode-of-inheritance in different environments. Only 5.2% of the 77 repeatable consensus QTL changed the direction of additive-effect, which suggested that the relative superiority of one allele over the others was stable in different environments. In contrast, 31.2% of the 77 repeatable consensus QTL changed their dominant-effect directions in different environments. In addition, only 20.8% ( = 16/77) of these repeatable consensus QTL showed significant interaction with the environment at P≤0.05, which was lower than that (67.3% = 150/223) of the non-repeatable ones (Table S3C). Therefore, the expression, direction and effect of QTL were all dependent on environmental conditions, which suggested the variability of QTL.

**Table 5:** Overview of epistatic interactions identified in reconstructed F_2_ population and three environments for 15 yield-correlated traits.

Trait	SY§	SP	BY	PN	HI	PH	PY	SN	BN	OIL	PRO	MT	FT	SW	DT	Total
Total number	17	29	15	19	13	22	18	21	19	15	16	13	17	28	10	272
Repeatable	2	0	0	0	0	0	0	0	0	0	0	0	0	0	0	2
*R^2^* min (%)	4.2	1.6	2.1	1.9	3.5	2.0	3.1	2.8	2.0	3	1.9	2.9	1.4	1.4	4.1	1.4
*R^2^* max (%)	9.1	10.0	9.6	18.3	7.9	8.3	16.6	12.5	14.5	15.6	13.9	9.0	14.2	11.8	9.7	18.3
*R^2^* mean (%)	5.8	4.5	4.3	5.0	5.8	4.5	6.0	5.0	5.0	6.4	5.0	6.3	5.1	3.6	6.8	5.1
NN type*	6	14	8	9	7	11	6	11	11	9	10	9	10	10	5	136
NS/SN type	9	10	6	9	4	6	8	9	7	5	4	4	5	13	4	103
SS type	2	5	1	1	2	5	4	1	1	1	2	0	2	5	1	33
Total *R^2^*_AA_mean (%)	14.8	20.5	18.5	13.5	16.5	13.1	15.0	14.2	14.4	16.1	13.3	15.4	15.2	12.8	10.8	14.6
Total *R^2^*_AD/DA_mean (%)	12.2	13.7	8.4	8.5	14.0	11.1	12.9	13.4	9.2	8.7	4.9	8.5	7.6	11.3	7.6	10.4
Total *R^2^*_DD_mean (%)	6.4	9.3	5.6	9.5	7.0	9.0	8.1	7.3	8.0	7.2	4.0	3.5	6.3	9.3	4.1	7.1
Total *R^2^*_E-QTL_mean (%)†	33.4	43.5	32.5	31.5	37.5	33.2	36.0	34.9	31.6	32.0	22.2	27.4	29.1	33.4	22.5	32.1
Total *R^2^*_M-QTL_mean (%)	26.4	36.0	27.5	28.3	35.2	30.1	35.7	34.0	25.5	51.7	30.4	36.2	48.9	53.7	36.8	36.0
Number of loci that involved one or multiple epistatic interactions																
One	12	19	10	13	3	21	8	11	14	13	10	10	6	16	7	173
Two	13	23	12	14	14	14	16	22	14	12	11	8	17	21	9	220
Three	2	9	2	6	4	6	5	6	6	4	4	4	7	11	2	78
Four	4	4	3	3	3	2	5	0	1	0	4	2	2	5	2	40
Five	1	1	2	1	2	1	2	2	2	0	3	0	1	2	0	20
Six	0	0	1	0	0	0	0	0	1	0	0	2	1	1	0	6
Seven	2	2	0	1	0	0	0	1	0	1	0	0	0	0	0	7

§ For the abbreviation of the traits (ordered according to their correlation coefficients with SY), see MATERIALS AND METHODS.

*Epistatic interactions between (SS) two loci with significant main-effects, (SN/NS) a locus with significant main-effect and a locus with non-significant main-effect, and (NN) two loci with non-significant main-effects.

† M-QTL and E-QTL are the abbreviations for main-effect QTL and epistatic QTL respectively.

The confidence intervals of most consensus QTL determined for each trait overlapped (Table S3D). The 300 consensus QTL for the 15 yield-correlated traits were therefore subjected to a second round of meta-analysis, which resulted in the integration of 220 consensus QTL into 84 pleiotropic unique QTL.

### Genome-wide detection and analysis of epistatic interactions in the reconstructed F_2_ population and three environments for 15 yield-correlated traits

**Figure 2:**
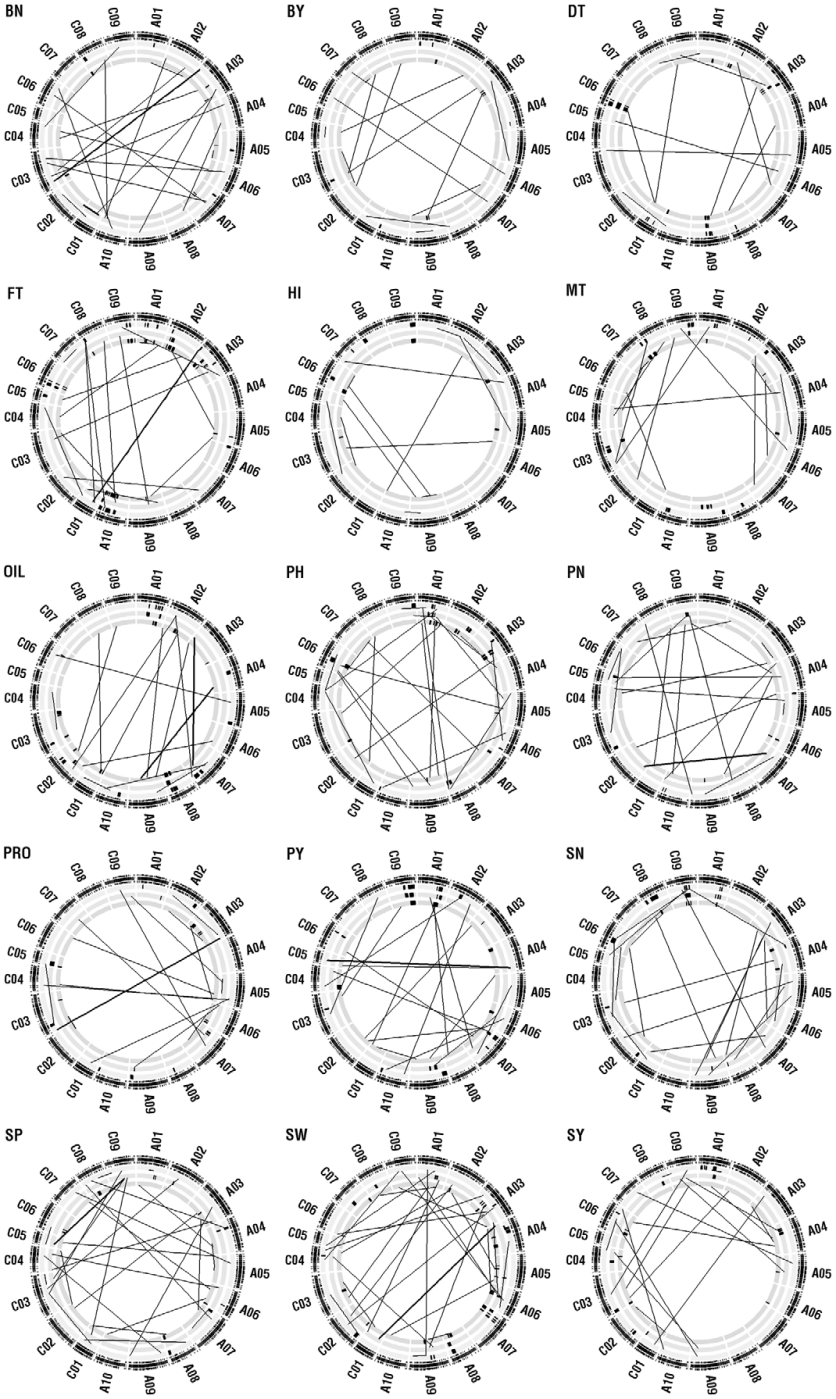
The genome-wide distribution of epistatic interactions identified in reconstructed F_2_ and three environments for each of the 15 yield-correlated traits. The TNDH linkage map was shown as a black circle (separated by a small gap) with vertical lines to indicate the position of the molecular markers, and around which the labels represent the names of the 19 linkage groups (A genome: A01-A10; C genome: C01-C09). The following three grey circles represent the three environments (from outside to inside, that is S6, S5 and N6 evinronment), on which The long black lines indicated the positions of the two loci involved in epistatic interactions and the width of the epistatic interaction line imply the magnitude of their phenotypic variance (*R* ^2^<10%; 10%≤*R* ^2^<20%). To illustrate the relationship of the positions of QTL and epistatic interactions, the QTL are also drawn with short curves where their lengths show the confidence interval and the circle indicates the peak position. The letters at the top left corner of these circles represent the abbreviation of each trait (see MATERIALS AND METHODS).

A total of 522 statistically significant epistatic interactions were identified for the 15 yield-correlated traits in two populations and three environments and most of them were also confirmed by the two-way analysis of variance (data not shown). Of these significant epistatic interactions, 272 were identified from the reconstructed F_2_ population (ranging from 11 to 29 for the different traits) (Table 5; Figure 2), potentially responsible for heterosis and were the objectives of the following analysis. Only two epistatic interactions of seed yield, which were detected in different environments and located in similar positions, were considered as repeatable, which suggested epistatic interactions of yield-correlated traits were extremely sensitive to the environmental variation. A total of 136, 103 and 33 epistatic interactions belonged to NN (the two loci involved in epistatic interaction were both with non-significant main-effects), NS (the two loci involved in epistatic interaction was one with significant main-effect and the other one with non-significant main-effect,) and SS (the two loci involved in epistatic interaction were both with significant main-effects) type of epistatic interactions respectively, which indicated most loci of epistatic interactions have no significant effect on trait performance alone but may affect it by epistatic interaction with other loci. The 272 epistatic interactions explained 1.4–18.3% (mean = 5.1%) of the phenotypic variance, while 95.6% showed only moderate effect, with *R* ^2^<10% (Table S4). It should be noted that 91.9% of the 272 epistatic interactions occurred between different chromosomes.

The proportion of the loci involved in multiple (2–7) epistatic interactions varied from 52.3% (for plant height) to 88.5% (for harvest index) for different traits and with a mean of 68.2% on average (Table 5), which indicated the prevalence of pleiotropic loci regulating heterosis on an epistatic level. For example, seven epistatic interactions (*eqOIL.13-16/14-26*, *eqPN.13-16/16-28*, *eqSN.11-42/13-16*, *eqSP.13-16/19-12*, *eqSP.11-14/13-16* **,** *eqSY.13-16/19-21*, and *eqSY.13-16/19-20*,) shared the common chromosome interval 13-16 indicating existence of a hotspot (Table S4).

### Mode-of-inheritance of QTL and epistatic interactions

**Figure 3:**
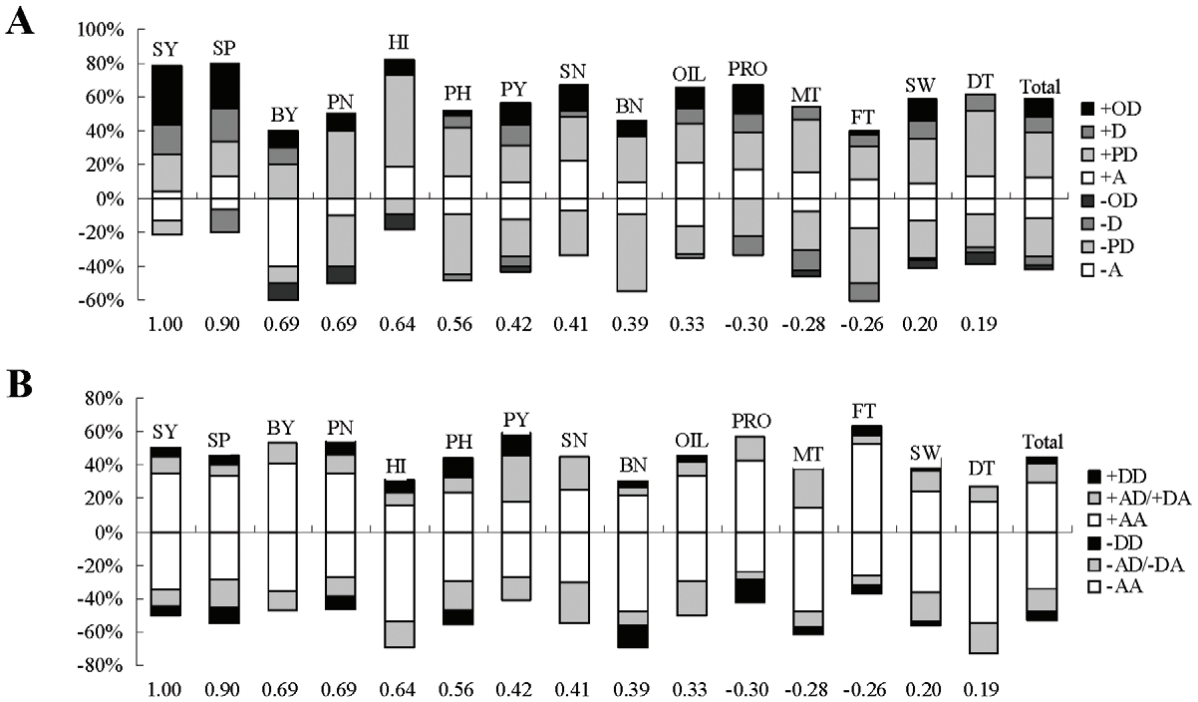
Distribution of qualitative mode-of-inheritance of QTL (A) and epistatic interactions (B) for 15 yield-correlated traits. Each vertical bar represents the proportion of QTL and epistatic interactions for each trait, colored according to mode-of-inheritance categories: A, additive; PD, partial-dominant; D, dominant; OD, over-dominant; AA, additive × additive; AD/DA, additive × dominant/dominant × additive; DD, dominant × dominant. The bars above and under the abscissa are respectively for the QTL and epistatic interactions with positive (+) and negative (−) genetic effect. The correlation coefficients between each trait and seed yield were indicated at the bottom.

Four kinds of QTL mode-of-inheritance (A; PD; D; OD) and three kinds of epistatic interactions mode-of-inheritance (AA; AD/DA; DD) were found for the 15 yield-correlated traits, which accounted for 24.6%, 49.0%, 13.8%,12.6%, and 63.0%, 26.0%, 11.0% respectively (Figure 3; Table 6). For the same trait, the QTL and epistatic interactions showed an unequal distribution among different mode-of-inheritance categories. For the same mode-of-inheritance category of QTL or epistatic interactions, unequal distribution was also observed among different traits, which suggested that the genetic mechanism underlying the heterosis of different traits might be different. Seed yield and seed number per plant clearly showed the highest proportion of +D/+OD mode-of-inheritance, which accorded well with the highest mid-parent heterosis of both traits. The dominant-effect direction of 41.8% QTL, 54.0% (48 out of 89, 48 from negative and 41 from positive) AD/DA and 48.7% (19 out of 39, 19 from negative and 20 from positive) DD epistatic-effect was negative, which was consistent with the low correlation between marker heterozygosity and mid-parent heterosis/hybrid performance.

**Table 6:** Comparison of qualitative mode-of-inheritance of QTL and epistatic interactions between different phenotypic categories.

Mode-of-inheritance§category		Sign	Fifteen yield-correlated traits				Nine metabolic traits	*P*_t-test_
			With heterosis	Without heterosis	*P*_t-test_	Total		
Identified QTL	A	-	11.2% (19)	12.7%(28)	0.834	12.1%(47)	22.1%(43)	0.197
		+	11.2% (19)	13.6%(30)	0.130	12.6%(49)	10.8%(21)	0.914
		*P*_t-test_	0.6024	0.293		0.923	0.105	
	PD	-	21.8%(37)	23.2%(51)	0.483	22.6%(88)	31.8%(62)	0.151
		+	27.1%(46)	25.9%(57)	0.682	26.4%(103)	26.7%(52)	0.650
		*P*_t-test_	0.156	0.250		0.079	0.287	
	D	-	2.9%(5)	6.4%(14)	0.123	4.9%(19)	2.1%(4)	0.122
		+	8.8%(15)	9.1%(20)	0.222	9.0%(35)	3.1%(6)	**0.048**
		*P*_t-test_	0.105	0.214		0.063	0.342	
	OD	-	2.4%(4)	2.3%(5)	0.843	2.3%(9)	3.1%(6)	0.565
		+	14.7%(25)	6.8%(15)	0.109	10.3%(40)	0.5%(1)	**0.000**
		*P*_t-test_	**0.002**	0.305		**0.003**	0.339	
	total	*-*	38.2%(65)	44.5%(98)		41.8%(163)	59.0%(115)	
		_+_	61.8%(105)	55.5%(122)		58.2%(227)	41.0%(80)	
Epistatic interactions	AA	-	33.6%(72)	35.0%(48)	0.8774	34.2%(120)	30.1%(53)	0.078
		+	28.5%(61)	30.7%(42)	0.7052	29.3%(103)	30.7%(57)	0.353
		*P*_t-test_	0.096	0.479		0.090	0.350	
	AD/DA	-	14.0%(30)	13.1%(18)	0.5892	13.7%(48)	12.5%(22)	0.423
		+	11.2%(24)	12.4%(17)	0.8892	11.7%(41)	10.2%(18)	0.705
		*P*_t-test_	0.362	0.955		0.463	0.802	
	DD	-	6.1%(13)	4.4%(6)	0.9934	5.49%(19)	2.8%(5)	0.335
		+	6.5%(14)	4.4%(6)	0.2891	5.7%(20)	8.0%(14)	0.253
		*P*_t-test_	0.485	0.708		0.752	0.859	0.078
	total	*-*	53.7%(115)	52.6%(72)		53.3%(187)	47.3%(80)	
		*+*	46.3%(99)	47.4%(65)		46.7%(164)	52.7%(89)	

§ The abbreviations of the Mode-of-inheritance categories. A: additive; PD: partial-dominant; D: dominant; OD: over-dominant; AA: additive × additive; AD/DA: additive × dominant/dominant × additive; DD: dominant × dominant.

To test whether the mode-of-inheritance of identified QTL and/or epistatic interactions was associated with the significance of heterosis, a *t* test was used for each mode-of-inheritance category between the nine traits with heterosis and the other six traits without heterosis and no significant differences were found (Table 6). However, between the 15 yield-correlated traits and 9 seed-quality/metabolic traits (glucosinolates, erucic acid, linolenic acid, linoleic acid, palmitic acid, oleic acid, stearic acid, α-tocopherol and γ-tocopherol contents in seeds, which were not significantly correlated with seed yield and unpublished in the current research), significant and extremely significant differences were found for +D and +OD mode-of-inheritance, respectively. In addition, for the nine traits with significant heterosis, the direction of OD effect was more frequently found to be positive than to be negative.

### Phenotypic effect of QTL and epistatic interactions

**Figure 4:**
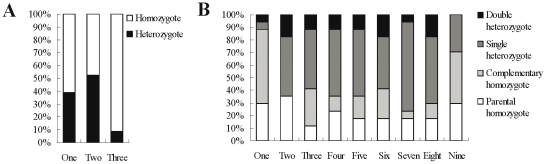
The relative performance of all kinds of genotypes of QTL (A) and epistatic interactions (B) for seed yield. The abscissa and ordinate respectively represents the relative place and the proportion of each type of genotype. The three genotypes of each QTL in reconstructed F_2_ population were classified into two types: homozygote and heterozygote. The nine genotypes of each epistatic interaction in reconstructed F_2_ population were classified into four types: parental homozygote, complementary homozygote, single heterozygote and double heterozygote.

To test the effect of identified QTL and epistatic interactions on the trait performance of the reconstructed F_2_ population for 15 yield-correlated traits, the performance of all kinds of genotypes was calculated (using the marker that was closest to the peak position of the identified QTL and epistatic interactions), compared and sorted. For the single-locus analysis, a homozygote was frequently the best and also the worst genotype, while a heterozygote was the most unlikely best and also worst genotype (Table S5A). For the two-locus analysis, a complementary homozygote (two loci were homologous for Tapidor and Ningyou7 respectively) was frequently the best genotype, followed by a parental homozygote (two loci were homologous for Tapidor or Ningyou7 respectively), a single heterozygote and a double heterozygote for almost all traits (Table S5B). For example in the case of seed yield, it was deduced that, in order to get the best genotype only 39.1% and 8.8% loci of identified QTL and epistatic interactions (21.1% for all loci involved) respectively, should be heterozygous (Figure 4). This accorded well with the previous finding that the seed yield of many lines in the reconstructed F_2_ population was higher than that of the F_1_ hybrid.

## Discussion

### Reconstructed F_2_ population is very suitable for heterosis study

The reconstructed F_2_ population used here holds several unique characteristics for dissecting the genetic architecture of heterosis. Firstly, it is well known that the F_2_ population was theoretically the most complete and informative source for most genetic analysis [12]. The genotype of the reconstructed F_2_ population was basically the same to that of the F_2_ population because the genotype of double haploid lines used in making the reconstructed F_2_ population was essentially the same as that of the gamete produced by the F_1_ hybrid (except for the possibility that genotypic selections existed in the process of microspore culture). In this sense, the reconstructed F_2_ population is more similar to the F_2_ population than the immortalized F_2_ population produced by the random intercross of recombinant inbred lines [13]. Secondly, each genotype of the reconstructed F_2_ population was represented by many individuals and thus permitted replicated experiments in multiple environments, so the reconstructed F_2_ population was better than the F_2_ and F_2:3_ populations. This also increased the power (or decreased experimental error) and reproducibility of QTL detection, and especially facilitated the analysis of environmental response of QTL in natural environments. Thirdly, additive, dominant and all kinds of epistatic effects (including AA, AD/DA and DD) can be well estimated in one population, thus increasing the accuracy of the estimation of dominant degree, mode-of-inheritance and especially the relative importance of all kinds of genetic effects in the expression of heterosis. Therefore, for heterosis study reconstructed F_2_ population is also better than BC, TC, NCIII and TTC populations in this sense. However, it should be noted that among all of the available experimental designs, TTC population has the unique potential to identify QTL that is directly linked to heterosis [14].

### Level of heterosis across traits and species

In all environments, seed yield showed the strongest heterosis among the 15 yield-correlated traits (Table 2), consistent with the findings in other rapeseed research [6],[7] as well as in other crops and plants, such as rice [8], [15], maize [9], [16], [17], [18], *Arabidopsis* [19], [20] and tomato [21]. This confirmed the hypothesis that complex traits usually express higher heterosis than component traits [22]. Interestingly, the theoretical mid-parent heterosis of seed yield [23] was calculated as: (1 + 18.4%) × (1 + 10.1%) × (1 + 2%) - 1 = 30.6%, a value which was very clear to the true value (31.4%) of mid-parent heterosis of seed yield (18.4%, 10.1% and 2% was the mid-parent heterosis mean in the reconstructed F_2_ population, respectively, for the three yield component traits). In addition, the yield heterosis of the tomato +/*sft* heterozygote could be traced back to component traits, number of flowers per plant and fruit weight [24]. This suggested that the heterosis of complex trait (such as yield) can be well explained by that of the component traits, because the middle and/or weak heterosis of the component traits may result in high heterosis of the complex traits in a multiplicative manner [23], [25],

Generally, the level of mid-parent heterosis for similar traits in the current research as well as other research in rapeseed [6], [7], rice [8], [15], wheat [26], *Arabidopsis* [19], [20] and tomato[21] were all much lower than that of the corresponding traits in maize [9], [16], [17], [18]. This may be attributable to differences in reproductive biology. Maize is an allogamous species and was supposed to have more deleterious alleles than autogamous species (because in autogamous species, deleterious alleles are possibly eliminated by natural and artificial selection since the individuals are homozygous), so the extent of inbreeding depression in maize was greater than that in rice, wheat, tomato and *Arabidopsis*, the autogamous species, and rapeseed, a partially allogamous crop [27], [28].

### Mode-of-inheritance of QTL and epistatic interactions

No significant difference was found for the proportion of the eight model-of-inheritance categories of QTL between the nine traits with heterosis and the other six traits without heterosis. This suggested that the presence or absence of heterosis was not associated with QTL mode-of-inheritance in the current research, which may be because the dominant effect only accounted for a small proportion of variance when compared with the epistatic effect of these traits (Table 5). However, between the 15 yield-correlated traits and the 9 seed-quality/metabolic traits, significant and extremely significant differences were found for +D and +OD mode-of-inheritance. This indicated +OD/+D mode-of-inheritance was associated with the traits of yield category, which may be because the occurrence of +OD/+D QTL for yield-correlated traits will increase crop productivity during the processes of domestication. Thus, OD may be an essentially pseudo-OD that involves linked loci with dominant alleles in repulsion [4], [5]. We detected A, PD and D QTL for both yield-correlated and seed-quality/metabolic traits, but OD was basically absent in seed-quality/metabolic traits. This indicated that pseudo-OD due to random linkage is unlikely to be the major genetic basis underlying OD QTL, and thus we favored the true OD model. In fact, +OD/+D QTL was prevalent in almost all research regarding the genetic basis of yield, life-history and reproductive traits in crops. In a tomato introgression line population, +OD QTL was more prevalent for the reproductive traits than nonproductive traits [21]. In a summary research, the dominance effect was found to be larger in life-history traits than in morphological traits [29]. Although only a few studies reported the QTL mapping of metabolic traits, the results all showed that only a few metabolic-QTL showed OD mode-of-inheritance [30], [31]. This suggested that different phenotypic classes may have different dominance relationships among variable alleles, possibly due to differences in the complexity underlying the molecular networks [32], [33]. More importantly, the sign of dominant-effect of OD QTL for the nine traits with heterosis was more frequently found to be positive than to be negative, which suggested that selection also has changed the frequency of the direction of OD effect for these traits of heterosis. This is understandable, since a positive OD effect may undoubtedly increase the heterosis and yield of hybrids.

However, no mode-of-inheritance categories and their direction of epistatic interactions showed significant difference in proportion among different phenotypic categories (Table 6). In fact, this phenomenon seemed to be typical in other crops. In a two-year experiment conducted in an “immortalized F_2_” population of an elite rice hybrid known as Shanyou63, the proportions of three kinds of epistatic interactions (AA, AD/DA and DD) were almost the same between reproductive (grain yield, tillers per plant, grains per panicle *etc.*) and non-reproductive (heading date, plant height and panicle length *etc.*) traits [34]. In a two-location experiment conducted in an F_2:3_ population in maize, no significant difference was also found in the proportion of three kinds of epistatic interactions between yield traits (such as grain yield, rows number, kernels per row *etc.*) and morphological (ear length, ear diameter and axis diameter *etc.*) traits [35]. This suggested that selection was not effectual at epistatic level during the domestication of rapeseed, as well as other crops. This was understandable: since epistatic interactions were more dependent on the genetic background and environmental variations than QTL [8], [36], their role was variable, and thus capturing the best gene combination(s) was difficult for breeders.

It should be noted that the relative proportion of the four kinds of mode-of-inheritance of QTL showed great differences in different traits and studies. For example, in the same QTL mapping experiment of nine yield traits, the predominant mode-of-inheritance of QTL was over-dominant and additive, in an intraspecific and intrasubspecific rice hybrid [15]. However, in all research in which the three kinds of epistatic effects could be resolved [13], [16], [37], AA interaction occurred at the highest frequency for all traits, followed by AD/DA and the DD interaction at the middle and lowest frequency, respectively. This confirmed that selection has great but little or no impact on mode-of-inheritance of QTL and epistatic interactions, respectively. In addition, in all cases the practical proportions (usually >50%, <40% and <8%) of AA, AD/DA and DD interactions were all quite different with their theoretical proportions of 25%, 50% and 25% [38], respectively. This provided the evidence that the identified epistatic interactions were absolutely not the results of chance events.

### Environmental response of QTL and epistatic interactions

The meta-analysis of QTL identified in different environments facilitated the exact estimation of the environmental response of QTL [39]. Totally, 74.3% (223) of the consensus QTL (Table 4) and 99.3% (270) of the epistatic interactions (Table 5) for the 15 yield-correlated traits was specifically identified in one of the three environments, which indicated the great impact of natural environments on the genes underlying the heterosis of these yield-correlated traits. These proportions were much higher than the corresponding ones (48.4% and 91.6%) of the other 9 seed-quality/metabolic traits (unpublished data), which accorded well with the broad-sense heritability of these traits. In fact, the high dependency on environment seemed to be a common character of the QTL and epistatic interactions for heterosis in other research. In a two year experiment conducted in an F_2:3_ population derived from an elite rice hybrid (Shanyou63), 62.5% QTL and 90.6% digenic interactions for grain yield and the three yield component traits were observed in only one year [38]. In another two year experiment conducted in an “immortalized F_2_” population derived from the same rice hybrid, 67.5% QTL and 91.5% digenic interactions for the same four yield traits were detected in only one year [13]. In a two-location experiment conducted in an F_2:3_ population derive from an elite maize hybrid, 62.1% QTL and 91.8% of digenic interactions for grain yield and the three yield component traits were detected in only one location [16]. It should be noted that the proportion of environment-specific epistatic interactions was much higher than that of QTL in all cases, which was understandable since the epistatic interactions involved two genetic loci which were also dependent on environmental conditions. It should also be noted that the proportions of environment-specific QTL and epistatic interactions in the current research as well as other rapeseed research [37] were all higher than that in rice [13], [38] and maize [16], possibly due to the genome plasticity of polyploids [40], [41]. This indicated the high variability and plasticity of the genetic architecture of heterosis in rapeseed.

Furthermore, of the 77 repeatable consensus QTL for 15 yield-correlated traits, 68.8% changed their mode-of-inheritance in different environments (Table S3C). This proportion was also much higher than that (46.9%) of the 9 seed-quality/metabolic traits (data not shown). This indicated that the relative importance of dominant vs additive effect of QTL of different phenotypic categories may have different sensitivity to environmental variations, possibly due to differences in the intrinsic mechanism of regulation. Interestingly, the additive-effect direction of the repeatable consensus QTL was usually the same in different environments, which was consistent with previous research [39], [42], [43]. This has great significance for genetics and crop breeding: since the relatively favorable alleles identified in one environment were usually relatively favorable in another environment, the actual effect of selection might be well ensured. From an evolutionary point of view, these retained alleles all experienced the processes of far-flung natural or artificial selection, and alleles that were adaptable to changed environments could be successfully retained. Whereas, 24 of the 77 repeatable consensus QTL changed their dominant-effect direction in different environments, this proportion (31.2%) was much higher than that (5.2%) of the additive-effect direction. Furthermore, for the other 53 repeatable consensus QTL with a consistent dominant-effect direction, 54.7% changed their mode-of-inheritance in different environments. For example, the mode-of-inheritance of *qSY.A1-5* was changed from +PD in N6 environment to +OD in an S5 environment. This indicated that the favorable heterozygote identified in one environment was not always favorable in another environment.

### Genetic architecture of heterosis in rapeseed and other species

Using a reconstructed F_2_ population (that has the maximum similarity to an F_2_ population), a multiple-environment experiment and a high-density linkage map, we identified hundreds of QTL and epistatic interactions responsible for the heterosis of 15 yield-correlated traits. Surprisingly, 92.8% identified QTL and 95.6% epistatic interactions explained <10% of variance (Table S3; Table S4). This indicated that heterosis of yield-correlated traits in this cross was mainly controlled by numerous loci with very little effect [15], [18], [20]. In addition, the maximum variances explained by individual QTL and epistatic interactions were 20.8% and 18.3% respectively. Therefore, the development of QTL and epistatic interactions near-isogenic lines [9], [44] toward fine-mapping and finally cloning the genes responsible for heterosis in this cross would be very challenging [5], [45], [46].

In contrast with the high variability of QTL and epistatic interactions, their most important feature was the high proportion (73.3% and 68.2% respectively) that co-localized at the genomic level (Table S3; Table S4). This accorded well with the comprehensive correlation of the mid-parent heterosis/hybrid performance among these yield-correlated traits. These co-localizations indicated the existence of pleiotropic loci regulating heterosis. In fact, most published fine-mapped QTL or genes identified for yield heterosis exhibit pleiotropic effects on at least one or multiple yield-correlated traits [24], [47], [48], [49], [50], [51]. Fifteen of the 21 consensus QTL of seed yield co-localized with other consensus QTL and 7 of them co-localized with more than two consensus QTL. This indicated that, in addition to pleiotropy, the effect of the QTL for seed yield could be a synthetic effect of several tightly-linked QTL of different yield-correlated traits. The multiple co-localized QTL might come from the different environments, which indicated that the environmental conditions contribute to the variability and plasticity of the QTL for seed yield. It should be noted that more than half of the loci of the QTL and epistatic interactions were clustered in several chromosomes (Figure 1; Figure 2).

Research from autogamous species, such as *Arabidopsis* [20], [52], rice [15], [38], [53], [54], [55] and barley [56], usually showed that epistasis played a more important role than main-effect as the genetic basis of heterosis. In contrast, data from allogamous crops, such as maize [9], [57], [58], exhibited the reverse result, demonstrating that main-effect is more important than epistasis. This is not surprising, since co-adapted gene complexes exhibiting favorable epistatic effects can be more easily maintained in autogamous species than in allogamous species [27], [59]. Therefore, it is reasonable for our result to show that epistasis was somewhat more important than main-effect as the genetic basis of heterosis in rapeseed (a partially allogamous crop with an out-crossing rate of 10-30%), and is consistent with other research in rapeseed [6], [7]. According to the theory of classical genetics, only D, AA and DD effect are the genetic components of mid-parent heterosis [14], [60]. Furthermore, the average |D| was smaller than the average |A|, and their ratios ranged from 0.40 (for branch number) to 0.73 (for seed yield) and with a mean of 0.51 (Table 5). This suggested that dominant effect only accounted for a minor proportion of *R* ^2^ of QTL, whereas, AA and DD effects explained a major proportion (67.1%) of *R* ^2^ of epistatic interactions. In conclusion, our research showed that epistasis (especially AA epistasis) was the major genetic basis of heterosis in rapeseed (*Brassica napus* L.).

### Implications for evolution and crop breeding

The two parents used in this study, Tapidor and Ningyou7, are the representative of two highly diverse gene pools, the European winter-type rapeseed gene pool and the Chinese semi-winter type rapeseed gene pool, both adaptable to their corresponding agro-ecological areas [61]. The proportion of positive (54.9%) and negative (45.1%) additive-effect was basically equal (Table 6), which indicates that both gene pools harboured alleles adaptable to other agro-ecological areas [39]. One hundred and three epistatic interactions showed significant positive AA interactions, which indicated co-adapted gene complexes retained during the evolution of rapeseed, a phenomenon also found in other species [62], [63], [64]. Oilseed rape (AACC, 2n = 38) originated from the natural hybridization of *Brassica rapa* (AA, 2n = 20) and *Brassica oleracea* (CC, 2n = 18) and the following chromosome doubling [65], both of which also experienced an evolutionary process of triploidization [66]. Therefore, each gene has an average of 6 copies in rapeseed. If these duplicated genes favorably interacted with each other, this would result in ectopic heterozygosis and the fixed heterosis in inbred lines [67] **.** In fact, many epistatic interactions identified in reconstructed F_2_ and DH populations occurred between homologous intervals/blocks (data not shown), which indicated the existence of fixed heterosis loci in rapeseed. Since a high-density linkage map together with detailed chromosome block information was available, it was possible to study the hypothesis of fixed heterosis and demonstrate its advantage in the evolution of polyploids using two-segment near-isogenic lines [44] chosen from the backcross progenies in our laboratory. One hundred and twenty epistatic interactions of the 15 traits showed significant and negative AA interactions, which indicated the complementary homozygote of these epistatic interactions tended to enhance fitness. This also suggested that complementary loci played an important role in the maintenance of genetic variation in the rapeseed population. Therefore, reserving the adapted genes and co-adapted gene complexes (including fixed heterosis loci) in per se gene pool while further pyramiding the favourable genes and gene combinations (including fixed heterosis loci) in another gene pool may be an effective strategy to further improve rapeseed conventional cultivars in both agro-ecological areas. Consistent with the findings in other research in rapeseed as well as other species, a considerable proportion of dominant effect (41.8%) and DD interactive effect (48.7%) was negative (Table 6), which indicated the general existence of hybrid weakness genes across species [68], [69]. This suggested that heterozygote was not always advantageous for the hybrid performance and mid-parent heterosis in rapeseed. This conclusion was also confirmed by the comparison of phenotypic effects of all kinds of genotypes both at the single and two locus level. Therefore, the knockout or substitution of hybrid weakness genes represents a new avenue to further improve hybrid cultivars. It should also be noted that 58.2% of dominant effect and 51.3% of DD interactive effect was positive, which indicated heterozygosis played an important role in the fitness of natural populations by providing a heterozygous advantage to buffer against recessive alleles and providing genetic plasticity to variable environmental conditions [5].

Although homozygotes of the detected QTL and epistatic interactions were usually the best genotypes in rapeseed [37] as well as in rice [13] and maize [16], [35], the proportion still needs to be well demonstrated. The most striking finding in this research is that to be the best hybrid, most heterozygous loci (83.2% in this experiment) of all QTL and epistatic interactions in hybrid F_1_ should be homozygous, which accorded well with the results that only 19.2% of QTL and 17.4% of epistatic interactions showed positive OD/D and DD/AD(DA) mode-of-inheritance respectively. This suggested that, in most cases, homozygotes were more advantageous for trait performance than heterozygotes. At first view, this conclusion seemed unbelievable, a truth usually neglected, is that, heterosis (usually defined as mid-parent heterosis) and hybrid performance are related but essentially two different concepts, because the latter is more complex and equal to the former plus the parental mean. The cryptic meaning is that a hybrid showing the strongest mid-parent heterosis for a given trait did not always exhibit the best per se manifestation of the same trait. Similarly, a heterozygote may enhance mid-parent heterosis value but decrease per se hybrid performance. Therefore, our conclusion is not intricate, and this has great significance for genetics and crop breeding. Because heterosis usually coincides with the genetic distance between parents [70], to maximize heterosis, breeders usually adopted parents with greater genetic distance, and as a result, the unadapted germplasm was also adopted in the hybrid breeding scheme. Therefore, the final result is that the breeders get the combinations of max heterosis but not the best hybrids. To avoid the occurrence of this embarrassing situation, we suggest an adapted germplasm with relatively large genetic distance would be a better choice in a hybrid breeding scheme. In addition, our result also suggested the utilization of the residual heterosis of inbred and backcross progenies (such as F_2_, F_3_ and BC_x_ *etc*) in rapeseed as well as other partially-allogamous and autogamous crops would be feasible, because the over-F_1_ phenomenon for yield and/or biomass was usually found in the subsequent inbred and backcross progenies even for elite hybrids [13], [20], [37].

This research revealed that epistasis played an important role in the genetic architecture of trait performance and heterosis in autogamous and partially-allogamous crops. The research also showed that epistasis is very sensitive to environment, and the epistatic effect varied from one environment to another, thus artificial selection seemed to have little or no effect on it, though it has proved to be effectual at the single-locus level (illustrated by the association between +OD/+D QTL and the traits of yield category, and between positive signs of OD effects and traits with heterosis). This suggested that while challenging, marker-assisted selection to significantly improve the heterosis/hybrid performance of yield traits in the aforementioned crops has great potential.

## Materials and Methods

### Design and development of a reconstructed F_2_ population

A double haploid (DH) population of 202 lines was developed by microspore culture from the F_1_ cross between Tapidor (an European winter-type rapeseed cultivar) and Ningyou7 (a Chinese semi-winter type rapeseed cultivar) and named as TNDH [61]. A reconstructed F_2_ population was made by making 101 crosses per round between pairs of DH lines randomly chosen from the 202 lines of the TNDH population. In the spring of 2004 and 2005, three and four rounds of crossing were made by hand emasculation and hand pollination, resulting in 303 and 404 crosses respectively.

### Field experiments and trait measurements

The two populations (TNDH and reconstructed F_2_), two parents (Tapidor and Ningyou7) and F_1_ (Tapidor × Ningyou7) were grown in 3 different environments (year-location combinations) in China (Table 7). The field planting followed a randomized complete block design with three replications. Each plot was 3.0 m^2^ with 30 plants in N6 and S6 environments and 4.0 m^2^ with 40 plants in S5 environments, with a distance of 40 cm between rows and 25 cm between individuals. The seeds were hand sown and the field management followed standard agricultural practice. Twelve representative individuals from the middle of each row in each plot were hand harvested from ground level at maturity.

**Table 7:** Field experiment design and traits investigated.

Environment*	Location and geographic feature	Rapeseed growing period	Investigated traits§
S5	Jiangling, E113°25′/N30°30′/40 m	Oct, 2004—May, 2005	BN, DT, FT, MT, OIL, PH, PN, PRO, PY, SN, SP, SW, SY
S6	Daye, E114°48′/N30°06′/100 m	Oct, 2005—May, 2006	BN, BY, DT, FT, HI, MT, OIL, PH, PN, PRO, PY, SN, SP, SW, SY
N6	Dali, E109°56′/N34°52′/800 m	Sep, 2005—Jun, 2006	BN, BY, DT, FT, HI, MT, OIL, PH, PN, PRO, PY, SN, SP, SW, SY

*The first letter represents the orientation of the location in China: Jiangling and Daye are in southern (S) China and Dali is in northern (N) China; the last letter represents the year of harvest.

§ For the abbreviation of the traits, see MATERIALS AND METHODS.

A total of 15 traits were investigated: (1) seed yield (SY, kg/ha), (2) biomass yield (BY, kg/ha), (3) pod number per plant (PN); (4) seed number per pod (SN); (5) seed weight/1000 seeds (SW, g); (6) flowering time (FT, days); (7) maturity time (MT, days); (8) plant height (PH, cm); (9) branch number (BN); (10) development time of seeds (DT, days), calculated from maturity time and flowering time by the formula, DT  =  MT - FT; (11) seed number per plant (SP), calculated from SY and SW by the formula, SP  = 10 × SY (kg/ha)/SW (g/1000); (12) pod yield/100 pods (PY), calculated from SN and SW by the formula, PW  =  SN × SW/10; (13) harvest index (HI), calculated from BY and SY by the formula, HI  =  SY/(SY + BY)); (14) protein content in seeds (PRO), (15) oil content in seeds (OIL).

Seed yield per plant was measured as the average dry weight of seeds of the harvested individuals in a plot. Biomass yield per plant was measured as the average total above-ground (except the seeds) dry weight of the harvested individuals in a plot. Pod number was the number of well-filled, normally developed pods on each harvested individual in a plot. Seed number per pod was the average number of well-filled seeds from 100 well-developed pods, sampled from the primary branch in the middle of the harvested individuals in a plot. Seed weight was the average dry weight of 1000 well-filled seeds from three replicate samples, taken from the mixed seeds of the harvested individuals in a plot. Flowering time was measured as the interval between the date of sowing and the date when the first flowers emerged on 50% of the plants in a plot. Maturity time was measured as the interval between the date of sowing and the date when pods on most of the plants in a plot were yellow. Plant height was the height of each harvested individual in a plot, measured from the base of the stem to the tip of the main shoot. Branch number was the number of branches arising from the main shoot of each harvested individual in a plot. The oil and protein content of seeds was measured by Near Infrared Spectroscopy (NIR) using standard methods [71].

### Statistical analysis

Year-location combinations were treated as independent environments. Environment was treated as a fixed effect while genotype (DH or reconstructed F_2_ lines) was treated as a random effect. The broad-sense heritability was calculated as: *h* ^2^  =  *σ* ^2^_g_/(*σ* ^2^_g_ + *σ* ^2^_ge_/n + *σ* ^2^_e_/nr). Where, *σ* ^2^_g_ is the genetic variance, *σ* ^2^_ge_ is the interaction variance of genotype with environment, *σ* ^2^_e_ is the error variance, n is the number of environments and r is the number of replications. The genetic correlation was calculated as: *r*_G_  = *cov*_Gxy_/(*σ* ^2^_Gx_ × *σ* ^2^_Gy_)^1/2^, where, *cov*_Gxy_, *σ* ^2^_Gx_ and *σ* ^2^_Gy_ were the genetic covariance and variance of the pair-wise traits respectively. The significance of each genetic correlation was determined using a *t* test of the correlation coefficients [72]. The estimation of variance and covariance components were obtained using an SAS GLM procedure. The mean value for three replications in each environment for both populations was used in subsequent QTL analysis for all traits. General heterozygosity was calculated as N_H_/(N_T_+N_N_+N_H_). N_T_, N_N_ and N_H_ were the number of markers with genotypes of Tapidor, Ningyou7 and both parents, respectively. Special heterozygosity was calculated using the same formula but the statistics were restricted to the marker that was significantly associated with phenotype (data not shown).

### Genetic linkage map

A total of 786 markers were mapped to the new linkage map generated with the TNDH population using JoinMap 3.0 (http://www.kyazma.nl/index.php/mc.JoinMap). This covered 19 chromosomes identified as A1–A10 and C1–C9, with an average distance of 2.7 cM between markers (Table S6). The threshold for goodness of fit was set to ≤5.0 with logarithm of the odds ratio (LOD) scores 1.0 and a recombination frequency<0.4. The order of the markers on the linkage map agreed well with our published maps [61], [73]. The genotype of each RC-F2 line was deduced from the corresponding genotype of their parents.

### Genome-wide detection of QTL, meta-analysis and test the result of QTL meta-analysis

QTL were detected by composite interval mapping [74] using WinQTL cartographer 2.5 software (http://statgen.ncsu.edu/qtlcart/WQTLCart.htm). The number of control markers, window size and walking speed were set to 5, 10 cM and 1 cM respectively. The default genetic distance (5 cM) was used to define a QTL in a specific experiment. The threshold of experiment wise error rate was determined by permutation analysis with 1000 repetitions [75]. LOD values corresponding to P = 0.05 were used for identifying “significant” QTL. To avoid missing QTL with very small effects, a lower LOD value corresponding to P≤0.50 was adopted for the presence of “suggestive” QTL [73]. The overlapping “suggestive” QTL and all the “significant” QTL were admitted and named as “identified-QTL”.

The dominant degree of an identified-QTL was defined as d/|a|. For mode-of-inheritance of identified-QTL the QTL was defined as additive (|d/a|<0.2), partially-dominant (0.2≤|d/a|<0.8), dominant (0.8≤|d/a|<1.2) and over-dominant (|d/a|≥1.2) [76].

Since QTL of the same traits or related ones detected in different experiments and mapped to the same region of a chromosome, might in fact be several estimations of the position of one single QTL, algorithms for QTL meta-analysis were used to estimate the number and positions of the meta-QTL underlying the analyzed QTL [77]. This approach, using the *Akaike* information criterion (AIC), provided the basis on which to determine the number of meta-QTL that best fitted the results on a given linkage group. It also grouped the QTL detected in the different experiments into classes that correspond to the same QTL and provided a consensus estimation of QTL positions. Computations were conducted using the *BioMercator2.1* software [78]. At present, the method used in this software cannot distinguish between models with more than four meta-QTL on the same linkage group. If the estimated number of meta-QTL is more than four, *Biomercator2.1* declares the most probable model as one with a number of meta-QTL equal to the number of the analyzed QTL. Then the *Delete* function of the software was used to select specific segments of a linkage group separated by regions with no QTL and separately apply QTL meta-analysis to these segments. The software also provides a method to calculate 95% confidence intervals for the meta-QTL:
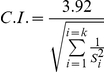
Where, *S_i_^2^* is the variance of position of the QTL_i_ and k is the total number of QTL integrated into the meta-QTL.

A two-round strategy of QTL meta-analysis was adopted. The QTL identified in different experiments were first integrated into consensus QTL, trait by trait. In the second round of QTL meta-analysis, the consensus QTL for the different traits was integrated into unique QTL.

To test the result of QTL meta-analysis, ANOVA implemented in SAS/Stat version 8e was utilized to identify QTL × environment interaction by GLM (generalized linear model) model: P  =  G + E + G × E. Where, P, G, E and G × E represent the phenotype and the effects of genotype, environment and genotype by environment interaction, respectively. The genotype of each consensus QTL was estimated by that of the molecular marker closest to it's peak position. The significant threshold was set as p≤0.05.

### Genome-wide detection of epistatic interactions

The maximum-likelihood estimation method in QTLmapper V2.0 software (http://www.cab.zju.edu.cn/ics/faculty/zhujun.htm) was employed to detect the epistatic interactions [79]. It was based on mixed linear model and performs composite interval mapping. The walking speed was set to 1 cM. The LR value corresponding to P = 0.005 was used as the threshold for claiming the presence of putative epistatic interactions. The significance of the epistatic effect was further tested by running the submenu of the Bayesian test (using P≤0.005).

## Supporting Information

Table S1 **Genetic correlations of the trait performance and mid-parent heterosis among the 15 investigated traits in three environments.**
(XLS)

Table S2 **ANOVA analysis and multiple comparison of trait performance of the two parents, F1 and two populations for 15 yield-correlated traits.**
(XLS)

Table S3 **The list of identified QTL, consensus QTL and unique QTL in three environments for 15 yield-correlated traits.**
(XLS)

Table S4 **The list of epistatic interactions identified from reconstructed F2 population in three environments for 15 yield-correlated traits.**
(XLS)

Table S5 **Comparison of the trait performance of genotypes for each identified QTL and epistatic interaction for 15 yield-correlated traits.**
(XLS)

Table S6 **A high-density linkage map of 786 molecular markers constructed with TNDH population.**
(XLS)

## References

[1] Shull GH (1908). The composition of a field of maize.. Ann Breed Assoc.

[2] Hua J, Xing Y, Wu W, Xu C, Sun X (2003). Single-locus heterotic effects and dominance by dominance interactions can adequately explain the genetic basis of heterosis in an elite rice hybrid.. Proc Natl Acad Sci U S A.

[3] Duvick DN (2001). Biotechnology in the 1930s: the development of hybrid maize.. Nat Rev Genet.

[4] Birchler JA, Yao H, Chudalayandi S (2006). Unraveling the genetic basis of hybrid vigor.. Proc Natl Acad Sci U S A.

[5] Lippman ZB, Zamir D (2007). Heterosis: revisiting the magic.. Trend Genet.

[6] Radoev M, Becker HC, Ecke W (2008). Genetic analysis of heterosis for yield and yield components in rapeseed (*Brassica napus* L.) by quantitative trait locus mapping.. Genetics.

[7] Basunanda P, Radoev M, Ecke W, Friedt W, Becker HC (2010). Comparative mapping of quantitative trait loci involved in heterosis for seedling and yield traits in oilseed rape (*Brassica napus* L.).. Theor Appl Genet.

[8] You A, Lu X, Jin H, Ren X, Liu K (2006). Identification of quantitative trait loci across recombinant inbred lines and testcross populations for traits of agronomic importance in rice.. Genetics.

[9] Frascaroli E, Cane MA, Landi P, Pea G, Gianfranceschi L (2007). Classical genetic and quantitative trait loci analyses of heterosis in a maize hybrid between two elite inbred lines.. Genetics.

[10] Peng J, Ronin Y, Fahima T, Roder MS, Li Y (2003). Domestication quantitative trait loci in Triticum dicoccoides, the progenitor of wheat.. Proc Natl Acad Sci U S A.

[11] Fu J, Keurentjes JJ, Bouwmeester H, America T, Verstappen FW (2009). System-wide molecular evidence for phenotypic buffering in Arabidopsis.. Nat Genet.

[12] Allard RW (1956). Formulas and tables to facilitate the calculation of recombination values in heredity.. Hilgardia.

[13] Hua J, Xing Y, Xu C, Sun X, Yu S (2002). Genetic dissection of an elite rice hybrid revealed that heterozygotes are not always advantageous for performance.. Genetics.

[14] Melchinger AE, Utz HF, Piepho HP, Zeng Z, Schon CC (2007). The role of epistasis in the manifestation of heterosis: a systems-oriented approach.. Genetics.

[15] Li L, Lu K, Chen Z, Mu T, Hu Z (2008). Dominance, overdominance and epistasis condition the heterosis in two heterotic rice hybrids.. Genetics.

[16] Yan J, Tang H, Huang Y, Zheng Y, Li J (2006). Quantitative trait loci mapping and epistatic analysis for grain yield and yield components using molecular markers with an elite maize hybrid.. Euphytica.

[17] Tang J, Yan J, Ma X, Teng W, Wu W (2010). Dissection of the genetic basis of heterosis in an elite maize hybrid by QTL mapping in an immortalized F_2_ population.. Theor Appl Genet.

[18] Flint-Garcia SA, Buckler ES, Tiffin P, Ersoz E, Springer NM (2009). Heterosis is prevalent for multiple traits in diverse maize germplasm.. Plos One.

[19] Meyer RC, Kusterer B, Lisec J, Steinfath M, Becher M (2010). QTL analysis of early stage heterosis for biomass in Arabidopsis.. Theor Appl Genet.

[20] Kusterer B, Muminovic J, Utz HF, Piepho HP, Barth S (2007). Analysis of a triple testcross design with recombinant inbred lines reveals a significant role of epistasis in heterosis for biomass-related traits in Arabidopsis.. Genetics.

[21] Semel Y, Nissenbaum J, Menda N, Zinder M, Krieger U (2006). Overdominant quantitative trait loci for yield and fitness in tomato.. Proc Natl Acad Sci U S A.

[22] Williams W (1959). Heterosis and the genetics of complex characters.. Nature.

[23] Zhang Q, Gao Y, Yang S, Ragab RA, Saghai Maroof MA (1994). A diallel analysis of heterosis in elite hybrid rice based on RFLPs and microsatellites.. Theor Appl Genet.

[24] Krieger U, Lippman ZB, Zamir D (2010). The flowering gene *SINGLE FLOWER TRUSS* drives heterosis for yield in tomato.. Nat Genet.

[25] Schnell FW, Cockerham CC (1992). Multiplicative vs arbitrary gene-action in heterosis.. Genetics.

[26] Singh H, Sharma SN, Sain RS (2004). Heterosis studies for yield and its components in bread wheat over environments.. Hereditas.

[27] Garcia AA, Wang S, Melchinger AE, Zeng Z (2008). Quantitative trait loci mapping and the genetic basis of heterosis in maize and rice.. Genetics.

[28] Springer NM, Stupar RM (2007). Allelic variation and heterosis in maize: how do two halves make more than a whole?. Genome Res.

[29] Roff DA, Emerson K (2006). Epistasis and dominance: evidence for differential effects in life-history versus morphological traits.. Evolution.

[30] Schauer N, Semel Y, Balbo I, Steinfath M, Repsilber D (2008). Mode of inheritance of primary metabolic traits in tomato.. Plant Cell.

[31] Lisec J, Steinfath M, Meyer RC, Selbig J, Melchinger AE (2009). Identification of heterotic metabolite QTL in *Arabidopsis thaliana* RIL and IL populations.. Plant J.

[32] Chan EK, Rowe HC, Kliebenstein DJ (2009). Understanding the evolution of defense metabolites in *Arabidopsis thaliana* using genome-wide association mapping.. Genetics.

[33] Schauer N, Semel Y, Roessner U, Gur A, Balbo I (2006). Comprehensive metabolic profiling and phenotyping of interspecific introgression lines for tomato improvement.. Nat Biotechnol.

[34] Hua J (2003). Genetic dissection on the basis of heterosis using an "immortalized F_2_" population..

[35] Yan J (2003). Study on the genetic basis of heterosis in maize and comparative genomics between rice and maize..

[36] Liao C, Wu P, Hu B, Yi K (2001). Effects of genetic background and environment on QTLs and epistasis for rice (*Oryza sativa* L.) panicle number.. Theor Appl Genet.

[37] Chen W (2008). Molecular dissection of genetic bases of important agronomic traits in oilseed rape Wuhan, China: Huazhong Agricultural University.

[38] Yu S, Li J, Xu C, Tan Y, Gao Y (1997). Importance of epistasis as the genetic basis of heterosis in an elite rice hybrid.. Proc Natl Acad Sci U S A.

[39] Shi J, Li R, Qiu D, Jiang C, Long Y (2009). Unraveling the complex tait of crop yield with quantitative trait loci mapping in *Brassica napus*.. Genetics.

[40] Dubcovsky J, Dvorak J (2007). Genome plasticity a key factor in the success of polyploid wheat under domestication.. Science.

[41] Sanjuan R, Elena SF (2006). Epistasis correlates to genomic complexity.. Proc Natl Acad Sci U S A.

[42] Maccaferri M, Sanguineti MC, Corneti S, Ortega JL, Salem MB (2008). Quantitative trait loci for grain yield and adaptation of durum wheat (*Triticum durum* Desf.) across a wide range of water availability.. Genetics.

[43] Li Z, Yu S, Lafitte H, Huang N, Courtois B (2003). QTL × environment interactions in rice. I. heading date and plant height.. Theor Appl Genet.

[44] Reif JC, Kusterer B, Piepho HP, Meyer RC, Altmann T (2009). Unraveling epistasis with triple testcross progenies of near-isogenic lines.. Genetics.

[45] Salvi S, Tuberosa R (2005). To clone or not to clone plant QTLs: present and future challenges.. Trend Plant Sci.

[46] Zhang Y, Luo L, Liu T, Xu C, Xing Y (2009). Four rice QTL controlling number of spikelets per panicle expressed the characteristics of single Mendelian gene in near isogenic backgrounds.. Theor Appl Genet.

[47] Xue W, Xing Y, Weng X, Zhao Y, Tang W (2008). Natural variation in *Ghd7* is an important regulator of heading date and yield potential in rice.. Nat Genet.

[48] Xing Y, Tang W, Xue W, Xu C, Zhang Q (2008). Fine mapping of a major quantitative trait loci, *qSSP7*, controlling the number of spikelets per panicle as a single Mendelian factor in rice.. Theor Appl Genet.

[49] Ni Z, Kim ED, Ha M, Lackey E, Liu J (2009). Altered circadian rhythms regulate growth vigour in hybrids and allopolyploids.. Nature.

[50] Terao T, Nagata K, Morino K, Hirose T (2010). A gene controlling the number of primary rachis branches also controls the vascular bundle formation and hence is responsible to increase the harvest index and grain yield in rice.. Theor Appl Genet.

[51] Guo M, Rupe M, Dieter J, Zou J, Spielbauer D (2010). *Cell Number Regulator1* affects plant and organ size in maize: implications for crop yield enhancement and heterosis.. Plant Cell.

[52] Melchinger AE, Piepho HP, Utz HF, Muminovic J, Wegenast T (2007). Genetic basis of heterosis for growth-related traits in Arabidopsis investigated by testcross progenies of near-isogenic lines reveals a significant role of epistasis.. Genetics.

[53] Mei H, Li Z, Shu Q, Guo L, Wang Y (2005). Gene actions of QTLs affecting several agronomic traits resolved in a recombinant inbred rice population and two backcross populations.. Theor Appl Genet.

[54] Luo L, Li Z, Mei H, Shu Q, Tabien R (2001). Overdominant epistatic loci are the primary genetic basis of inbreeding depression and heterosis in rice. II. Grain yield components.. Genetics.

[55] Li Z, Luo L, Mei H, Wang D, Shu Q (2001). Overdominant epistatic loci are the primary genetic basis of inbreeding depression and heterosis in rice. I. Biomass and grain yield.. Genetics.

[56] Xu S, Jia Z (2007). Genomewide analysis of epistatic effects for quantitative traits in barley.. Genetics.

[57] Lu H, Romero-Severson J, Bernardo R (2003). Genetic basis of heterosis explored by simple sequence repeat markers in a random-mated maize population.. Theor Appl Genet.

[58] Stuber CW, Lincoln SE, Wolff DW, Helentjaris T, Lander ES (1992). Identification of genetic factors contributing to heterosis in a hybrid from two elite maize inbred lines using molecular markers.. Genetics.

[59] Allard RW (1988). Genetic changes associated with the evolution of adaptedness in cultivated plants and their wild progenitors.. Heredity.

[60] Zeng Z, Wang T, Zou W (2005). Modeling quantitative trait loci and interpretation of models.. Genetics.

[61] Qiu D, Morgan C, Shi J, Long Y, Liu J (2006). A comparative linkage map of oilseed rape and its use for QTL analysis of seed oil and erucic acid content.. Theor Appl Genet.

[62] Ford-Lloyd BV, Newbury HJ, Jackson MT, Virk PS (2001). Genetic basis for co-adaptive gene complexes in rice (*Oryza sativa* L.) landraces.. Heredity.

[63] Li Z, Pinson SRM, Park WD, Paterson AH, Stansel JW (1997). Epistasis for three grain yield components in rice (*Oryza sativa* L).. Genetics.

[64] Matioli SR, Templeton AR (1999). Coadapted gene complexes for morphological traits in Drosophila mercatorum. two-loci interactions.. Heredity.

[65] U N (1935). Genome analysis in Brassica with special reference to the experimental formation of *B. napus* and peculiar mode of fertilization.. Japan J Bot.

[66] Lysak MA, Cheung K, Kitschke M, Bures P (2007). Ancestral chromosomal blocks are triplicated in Brassiceae species with varying chromosome number and genome size.. Plant Physiol.

[67] Abel S, Möllers C, Becker HC (2005). Development of synthetic *Brassica napus* lines for the analysis of “fixed heterosis” in allopolyploid plants.. Euphytica.

[68] Rhode JM, Cruzan MB (2005). Contributions of heterosis and epistasis to hybrid fitness.. The American Naturalist.

[69] Jiang W, Chu S, Piao R, Chin J, Jin Y (2008). Fine mapping and candidate gene analysis of *hwh1* and *hwh2*, a set of complementary genes controlling hybrid breakdown in rice.. Theor Appl Genet.

[70] East EM (1936). Heterosis.. Genetics.

[71] Mika V, Tillmann P, Koprna R, Nerusil P, Kucera V (2003). Fast prediction of quality parameters in whole seeds of oilseed rape (*Brassica napus* L.).. Plant Soil Environ.

[72] Kong F (2005). Quantitaive genetics in plants..

[73] Long Y, Shi J, Qiu D, Li R, Zhang C (2007). Flowering time quantitative trait Loci analysis of oilseed brassica in multiple environments and genomewide alignment with Arabidopsis.. Genetics.

[74] Zeng Z (1994). Precision mapping of quantitative trait loci.. Genetics.

[75] Churchill GA, Doerge RW (1994). Empirical threshold values for quantitative trait mapping.. Genetics.

[76] Stuber CW, Edwards MD, Wendel JF (1987). Molecular marker-facilitated investigations of quantitative trait loci in maize. II. factors influencing yield and its component traits.. Crop Sci.

[77] Goffinet B, Gerber S (2000). Quantitative trait loci: a meta-analysis.. Genetics.

[78] Arcade A, Labourdette A, Falque M, Mangin B, Chardon F (2004). BioMercator: integrating genetic maps and QTL towards discovery of candidate genes.. Bioinformatics.

[79] Wang D, Zhu J, Li Z, Paterson AH (1999). Mapping QTLs with epistatic effects and QTL × environment interactions by mixed linear model approaches.. Theor Appl Genet.

